# Endoscopic ultrasound-guided placement of fiducial markers for stereotactic body radiation therapy of pancreatic metastases from renal cell carcinoma

**DOI:** 10.1055/a-2109-0300

**Published:** 2023-07-13

**Authors:** Marcin Polkowski, Konrad Pawlewicz, Krzysztof Skoczylas, Ewa Wrońska, Małgorzata Lenarcik, Jarosław Reguła

**Affiliations:** 1 Department of Gastroenterology, Hepatology and Clinical Oncology, Center of Postgraduate Medical Education, Warsaw, Poland; 2 Department of Oncological Gastroenterology, The Maria Skłodowska-Curie National Research Institute of Oncology, Warsaw, Poland; 3 Department of Radiotherapy, The Maria Skłodowska-Curie National Research Institute of Oncology, Warsaw, Poland; 4 Department of Pathology and Laboratory Medicine, The Maria Skłodowska-Curie National Research Institute of Oncology, Warsaw, Poland

Isolated pancreatic metastases from renal cell carcinoma (RCC) have traditionally been treated with surgery or systemic therapy 1 . More recently, focal therapies such as endoscopic ultrasound (EUS)-guided radiofrequency ablation or stereotactic body radiation therapy (SBRT) have been reported in selected patients 2 3 4 . SBRT uses precision technology to accurately deliver high doses of radiation in a few treatment fractions to the tumor volume, while sparing the surrounding healthy tissues. Many SBRT protocols require the placement of fiducial markers in or near the tumor to facilitate accurate targeting 4 5 . Here we report EUS-guided fiducial placement for SBRT of pancreatic metastases in a 75-year-old woman with a history of left nephrectomy for RCC 20 years previously and a recent finding of two hypervascular pancreatic tumors on computed tomography (CT) scanning.

The procedure started with EUS examination of the entire pancreas, which revealed the two hypoechoic, well-delineated, well-vascularized tumors of 9 × 8 mm and 22 × 21 mm in size, in the pancreatic head and tail, respectively ( Video 1 ). Fine-needle biopsy (FNB) of the tumor in the tail was performed to confirm the diagnosis of RCC metastasis. Following FNB, a 0.018-inch platinum fiducial marker (LumiCoil; Boston Scientific) ( Fig. 1 ) was backloaded into a 22G needle (EZShot 3 plus; Olympus Europe) through the tip of the needle. After the tumor had been punctured and the needle tip positioned in its center under EUS guidance, the fiducial was deployed by pushing it out of the needle with the needle stylet. The same technique was used to deploy a second fiducial in the pancreatic head tumor ( Fig. 2 ). No adverse events were observed. Both fiducials were clearly visible and correctly positioned on imaging used for planning and the delivery of irradiation ( Fig. 3 and Fig. 4 ). On follow-up 1 year later, the pancreatic metastases were stable; however, pulmonary metastases were detected, and systemic treatment was therefore started.

**Video 1** Endoscopic ultrasound-guided placement of fiducial markers for stereotactic body radiation therapy of pancreatic metastases from renal cell carcinoma.


**Fig. 1:**
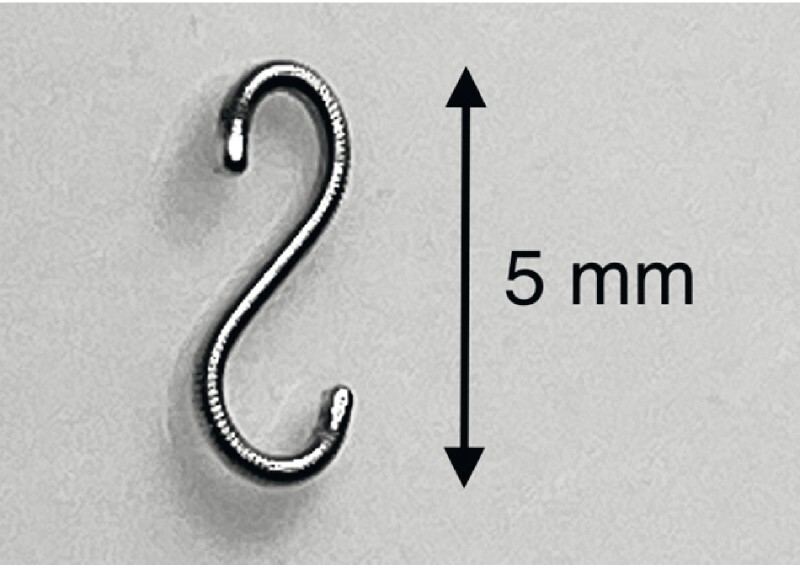
Photograph of the platinum fiducial marker (LumiCoil), which is 10 × 0.46 mm (0.018 inch) in size, can be loaded into a 22G endoscopic ultrasound needle, and coils into a figure eight-like configuration upon deployment.

**Fig. 2:**
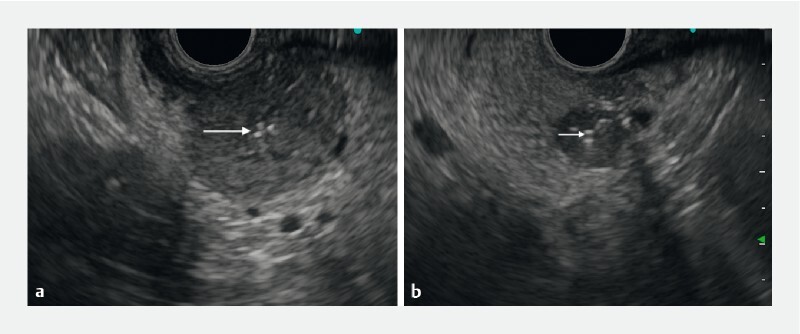
Endoscopic ultrasound (EUS) images showing the fiducial markers, visible as bright echoes (arrows) in the center of pancreatic metastases from renal cell carcinoma, which are seen as hypoechoic tumors in the: **a** pancreatic tail (22 × 21 mm in size); **b** pancreatic head (9 × 8 mm in size).

**Fig. 3:**
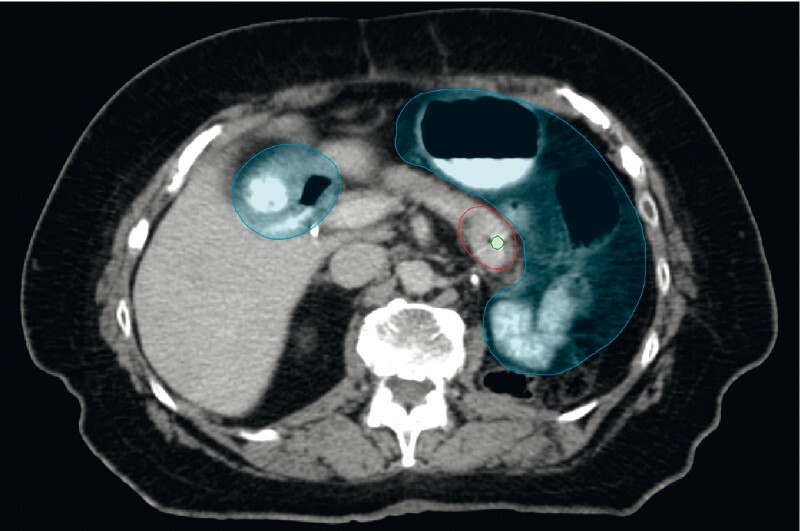
Computed tomography image used for radiotherapy planning showing the fiducial marker (encircled in green) implanted in the tumor of the pancreatic tail (encircled in red). Organs at risk (stomach, bowel) are marked in blue.

**Fig. 4:**
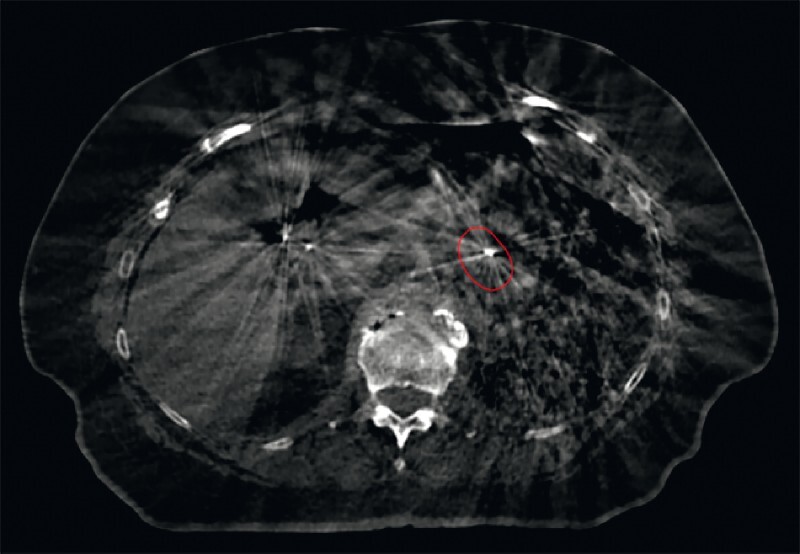
Cone-beam computed tomography (CBCT) image showing the fiducial marker implanted in the tumor of the pancreatic tail (encircled in red). The tumor itself is not visible, because of the low soft tissue contrast resolution of CBCT imaging; the radiopaque marker is essential for target volume localization. CBCT is performed before delivery of each radiation fraction to verify the target position.

Endoscopy_UCTN_Code_TTT_1AS_2AD

## Notes

Endoscopy E-Videos

https://eref.thieme.de/e-videos
*E-Videos* is an open access online section of the journal *Endoscopy* , reporting on interesting cases and new techniques in gastroenterological endoscopy. All papers include a high-quality video and are published with a Creative Commons CC-BY license. Endoscopy E-Videos qualify for HINARI discounts and waivers and eligibility is automatically checked during the submission process. We grant 100% waivers to articles whose corresponding authors are based in Group A countries and 50% waivers to those who are based in Group B countries as classified by Research4Life (see: https://www.research4life.org/access/eligibility/ ). This section has its own submission website at https://mc.manuscriptcentral.com/e-videos
